# Assessing the Implementation of Electronic Kidney Transplant Referral and Communication Programs

**DOI:** 10.1016/j.ekir.2026.106352

**Published:** 2026-02-10

**Authors:** Emma Blythe, Laura McPherson, Stephen Pastan, Rachel E. Patzer, Megan Urbanski

**Affiliations:** 1 Emory Health Services Research Center, Emory University School of Medicine, Atlanta, Georgia, USA; 2 Department of Epidemiology, Rollins School of Public Health, Emory University, Atlanta, Georgia, USA; 3 Center for Research and Evaluation, Kaiser Permanente Health Plan of Georgia, Inc., Atlanta, Georgia, USA; 4 Division of Renal Medicine, Department of Medicine, Emory University School of Medicine, Atlanta, Georgia, USA; 5 Regenstrief Institute, Indianapolis, Indiana, USA; 6 Department of Surgery, Indiana University School of Medicine, Indianapolis, Indiana, USA; 7 Division of Transplantation, Department of Surgery, Emory University School of Medicine, Atlanta, Georgia, USA

**Keywords:** dialysis, end-stage kidney disease, health communications, implementation science, kidney transplantation, mixed methods research, process evaluation

## Abstract

**Introduction:**

Efficient communication between dialysis and kidney transplant centers is crucial to move patients through the transplant process. The implementation outcomes of electronic transplant referral and communication programs to improve this communication are unknown.

**Methods:**

Using a convergent mixed-methods design, a process evaluation was conducted in 2023 to assess the dialysis and transplant center staff implementation of electronic transplant referral and communication programs according to the Reach, Effectiveness, Adoption, Implementation, and Maintenance (RE-AIM) framework via surveys and interviews.

**Results:**

Overall, 101 dialysis and 30 transplant center staff completed the survey, and 84% reported using electronic transplant referral and communication programs. Respondents were 96% female, 67% social workers, and 38% had > 10 years of field experience. Respondents indicated that electronic referral programs were easy to use (70%) and effective for sending or receiving transplant referrals (80%); however, unreliable use by other staff was a barrier to using these programs (36%). Interviews (*n* = 13) revealed that facilitators of the programs' use are multilevel and should involve leadership buy-in and comprehensive program training. Barriers to program use included an imbalance between program training costs and gains in efficiency, programs’ benefits limited by staff implementation, and an insufficient number of staff trained to use these programs.

**Conclusion:**

Electronic transplant referral and communication programs may streamline kidney transplant referrals and enhance communication; however, implementation varies between dialysis and transplant center staff. Understanding the implementation of these programs can inform interventions to refine and scale-up their use, to ultimately improve access to kidney transplant.

## Graphical abstract

**Figure:**
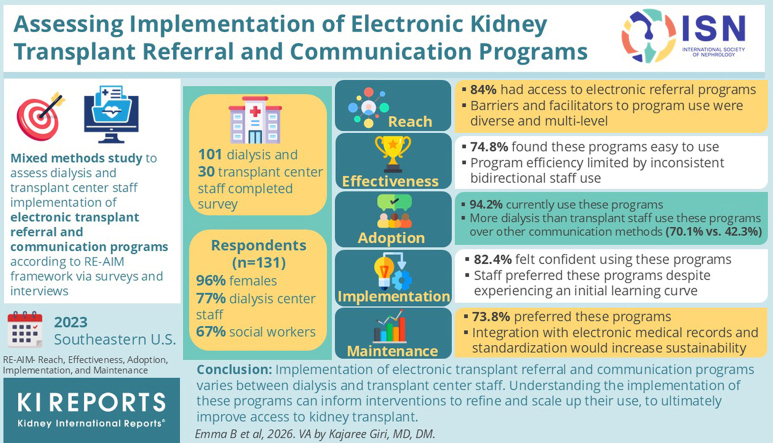

Although kidney transplant can improve survival and quality of life for many patients with end-stage kidney disease, the fragmented multistep transplant process inhibits transplant access (Figure 1).^1^^,^^2^ Health inequities exist throughout the process, often driven by nonmedical barriers and social determinants of health. For instance, Black patients, those with low educational attainment, or low socioeconomic status are less likely to be referred or evaluated for kidney transplant.^3^, ^4^, ^5^, ^6^ Although previous research indicates that kidney transplant barriers are multilevel and multifactorial,^7^, ^8^, ^9^ many interventions to increase transplant access are focused at the patient level rather than health system levels, where a greater impact may be achieved.^10^, ^11^, ^12^ For example, dialysis and transplant center staff have identified antiquated communication tools (e.g., fax) and the communication gap they create as major barriers to effective transplant care, highlighting the need for upstream quality interventions to improve communication and promote access to this life-saving treatment.^13^

**Figure 1:**
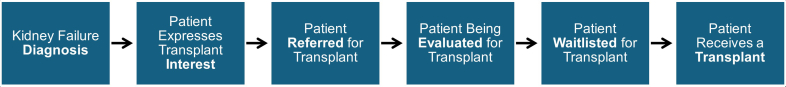
Stages of the kidney transplantation process for patients with end-stage kidney disease.

One attempt to bridge this communication gap has been the development of electronic kidney transplant referral and communication programs. These programs virtually connect dialysis and transplant centers, facilitating real-time, bidirectional communication across traditionally siloed health systems. However, their implementation has not been formally studied. Through integrating survey and interview data, we conducted a mixed-methods process evaluation of electronic kidney transplant referral and communication programs among dialysis and transplant center staff in the Southeastern US.

## Methods

### Study Design, Participants, and Data Collection

**Figure 2:**
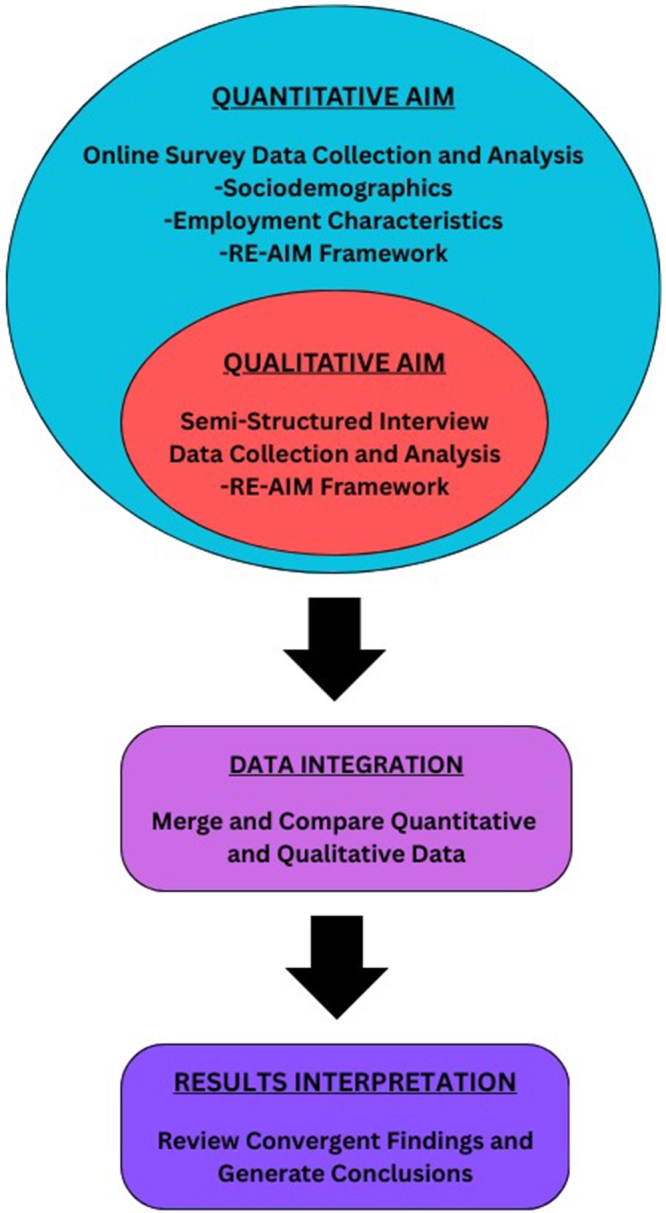
Nested convergent parallel mixed-methods study design.^14^ RE-AIM, Reach, Effectiveness, Adoption, Implementation, and Maintenance.

A nested convergent parallel mixed-methods process evaluation was conducted to evaluate the implementation of electronic transplant referral and communication programs among dialysis and transplant centers located in the Southeastern US (Figure 2).^14^ Web-based cross-sectional survey and semistructured telephone interview data were collected from May to October 2023. Quantitative and qualitative results of this study were individually analyzed, given equal priority, compared, and merged in mixed-methods analysis.^14^

Eligible participants were English-speaking adults (aged ≥ 18 years) currently employed at a dialysis or kidney transplant center in the US in Georgia, North Carolina, or South Carolina. Participants who were responsible for using electronic referral programs for referral or communication purposes, if they had been implemented in their facility were recruited from dialysis and transplant centers.

**Figure 3:**
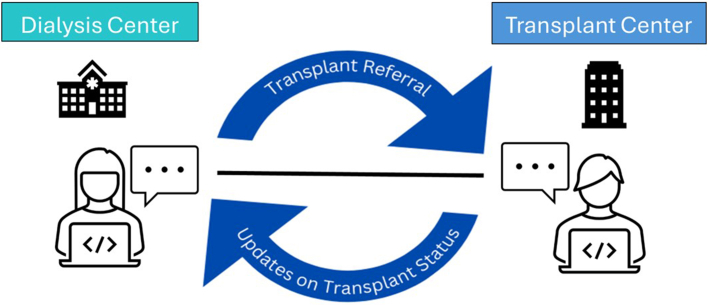
Visual of bidirectional communication between dialysis and transplant staff via an electronic transplant referral and communication program.

Electronic transplant referral and communication programs are online platforms that aim to enhance communication across health care settings, electronically manage patient care throughout the kidney transplant process, and facilitate real-time messaging and coordination between dialysis and transplant center staff. Within the online platforms, staff can upload transplant referrals, edit patient information, and securely message corresponding dialysis or transplant centers (Figure 3). These programs entered the commercial market approximately 10 years ago and are optional to implement for dialysis and transplant centers.

Survey respondents were recruited via professional listservs (e.g., Council of Nephrology Social Workers); email invitation from the Island Peer Review Organization End-Stage Renal Disease Network 6; and modified snowball sampling, where potential study participants were encouraged to share the email invitation with colleagues. Interview participants were purposively sampled from interested survey participants to capture a range of experiences within the study population (i.e., a range of staff types and roles, years of experience, geography, and survey responses). Survey data were captured and managed via Research Electronic Data Capture, a secure, web-based software platform.^15^^,^^16^ Interviews were conducted until saturation was reached, which were monitored by the study analysts. Participants provided online and/or verbal informed consent for their participation in the survey and interview. The survey and interview participants received $10 and $40, respectively, for their participation.

This study adhered to the Consolidated criteria for Reporting Qualitative research guidelines,^17^ the Strengthening the Reporting of Observational Studies in Epidemiology guidelines,^18^ and the checklist of mixed-methods elements in a submission to advance the methodology of mixed-methods research (Supplementary Table S1–S3).^19^ This study received approval from the Emory University Institutional Review Board (STUDY00004162).

### Study Variables and Measurements

The assessment of implementation outcomes was guided by the RE-AIM framework.^20^ Study instruments were informed by previous literature and subject matter experts and were mapped to the RE-AIM framework (Supplementary Table S4).^21^^,^^22^ Survey-specific measures included respondent sociodemographic characteristics (e.g., self-reported race) and employment-related characteristics (e.g., place of employment [dialysis or transplant center]). Study instruments were pilot-tested among colleagues with expertise in dialysis, kidney transplantation, and implementation science to assess face validity and were iteratively refined.

### Data Analysis

#### Surveys

Quantitative survey data were summarized using descriptive statistics. Quantitative survey items were compared by staff type (dialysis vs. transplant staff) using chi-square or Fisher exact tests. Open-ended survey items underwent a qualitative content analysis, where responses were coded and categorized. Results of the content analysis were reported using descriptive statistics.^23^ SAS version 9.4 was used for all quantitative data management and statistical analyses.^24^

#### Interviews

Semistructured interviews were conducted via telephone, audio-recorded, and transcribed verbatim. All transcripts underwent thematic analysis, which began with 2 researchers (EB and MU), trained in qualitative methods, reading line-by-line and creating analytic memos.^25^ A codebook was developed deductively from the RE-AIM framework and interview guide, and inductively from iterative transcript review and data extraction.^21^^,^^22^ Qualitative analysis of the interviews was based on the resulting codebook and relied on sufficient intercoder agreement (EB and MU) being met, before independent coding of transcripts. Coded segments were reviewed collectively to describe larger themes emerging from each RE-AIM dimension. Comparisons between dialysis and transplant staff interviews were made, and any notable discrepancies were reported by theme. Qualitative data were managed via MAXQDA 2022.^26^

#### Mixed-Methods Analysis

Following the individual analyses of the survey and interview data, a mixed-methods analysis was conducted, and results were systematically integrated via side-by-side joint displays that matched quantitative survey measures with qualitative interview themes.^14^ Quantitative and qualitative results were compared, contrasted, and integrated to generate mixed methods meta-inferences.^14^

## Results

### Survey Respondent and Interview Participant Characteristics

A total of 209 individuals were screened for the online survey; 59 were ineligible, 14 did not provide consent, and 5 completed less than half the survey, resulting in 131 total respondents (completion rate = 62.7%). Respondents were mostly female (*n* = 126, 96.2%), White (*n* = 65, 49.6%), and aged between 45 and 64 years (*n* = 63, 48.1%). Most survey respondents were dialysis center staff (*n* = 101, 77.1%), social workers (*n* = 88, 67.2%), and had > 10 years’ experience (*n* = 50, 38.2%). Most survey respondents reported using an electronic transplant referral and communication program (*n* = 110, 84.0%) (Table 1).

**Table 1:** Survey respondent characteristics

Demographic characteristics, *n* (%)	Total, *N* = 131
Primary place of work	
Dialysis facility	101 (77.1)
Transplant center	30 (22.9)
Number of facilities/clinics covered	
1	55 (42.0)
2	56 (42.8)
3	10 (7.6)
4+	8 (6.1)
Missing	2 (1.5)
Gender	
Female	126 (96.2)
Male	5 (3.8)
Race	
Asian	1 (0.8)
Black/African American	62 (47.3)
White	65 (49.6)
Prefer not to answer	2 (1.5)
Missing	1 (0.8)
Ethnicity	
Hispanic or Latino/a/x	4 (3.1)
Not Hispanic or Latino/a/x	124 (94.7)
Prefer not to answer	2 (1.5)
Missing	1 (0.8)
Age, yr	
18–34	18 (13.7)
35–44	39 (29.8)
45–64	63 (48.1)
65+	10 (7.6)
Prefer not to answer	1 (0.8)
State	
Georgia	74 (56.5)
North Carolina	40 (30.5)
South Carolina	17 (13.0)
Role	
Administrative assistant	4 (3.1)
Dietitian	1 (0.8)
Facility administrator	7 (5.3)
Medical director	1 (0.8)
Nephrologist	1 (0.8)
Nurse	1 (0.8)
Nurse coordinator	13 (9.9)
Nurse manager	12 (9.2)
Social worker	88 (67.2)
Other	3 (2.3)
Number of yrs worked in the field (dialysis/transplant)	
< 1	7 (5.34)
1–5	39 (9.8)
6–10	35 (26.7)
> 10	50 (38.2)
Use an electronic referral program	
Yes	110 (84.0)
No	21 (16.0)
Number of electronic referral programs used at facility/center^a^	
1	60 (54.6)
2	36 (32.7)
3	6 (5.5)
> 3	1 (0.9)
Missing	7 (6.4)

PA, physician assistant.

a Percentages calculated from number of respondents who reported using an electronic referral program (*n* = 110).

Of the 131 survey respondents, 13 dialysis and transplant center staff participated in semi-structured interviews, lasting approximately 45 minutes on average. The characteristics of interview participants are presented in Table 2.

**Table 2:** Interview participant characteristics

Characteristic, *n* (%)	Total, *N* = 13
Facility type	
Dialysis	10 (76.9)
Transplant	3 (23.1)
Role	
Nephrologist	1 (7.7)
Nurse manager	1 (7.7)
Social worker	10 (76.9)
Transplant specialist	1 (7.7)
Number of yrs of experience	
1–5	4 (30.8)
6–10	4 (30.8)
> 10	5 (38.5)
Gender	
Female	13 (100.0)
State	
Georgia	9 (69.2)
North Carolina	2 (15.4)
South Carolina	2 (15.4)
Race	
Black/African American	5 (38.5)
White	8 (61.5)
Ethnicity	
Hispanic/Latino/a/x	2 (15.4)
Not Hispanic/Latino/a/x	11 (84.6)
Number of electronic referral programs used at facility/center	
1	8 (61.5)
2	5 (38.5)

### Reach

#### Reach Survey Results

Of the 110 survey respondents (84.0%) who reported using an electronic transplant referral and communication program, most used only 1 program at their center (*n* = 60, 54.6%). Respondents most often learned about programs from their clinic supervisor or manager (*n* = 42, 38.2%), or a facility in-service learning session (*n* = 43, 39.1%). Training modality differed by staff type, with dialysis staff more likely to receive virtual training (65.9% vs. 42.9%) and transplant staff more likely to report no training (65.9% vs. 42.9%) (Table 3). Responses about why certain programs were used more often indicated that they were easy to use (*n* = 13, 37.1%) and widely adopted by other dialysis and transplant centers with mutual patients (*n* = 8, 22.9%) (Table 3).

**Table 3:** Reach: Survey and interview findings joint display

Qualitative theme and illustrative quotation	Quantitative survey item^a^	Meta-inferences
*Theme 1*: Facilitators of the program use are multilevel and include buy-in and endorsement from leadership, availability of program training, demonstrations of program value, and hands on interaction with the program. *“I think always take them up on the free demo. Most of these platforms will offer a free of charge demo of what the platform looks like and what it can do. So at least being open to seeing how it works I think is kind of step one, and then also I think—in transplant we’re a community, and I think maybe talking to other centers that have used it to understand the pros and cons and like, you know, what’s been your biggest benefit? What’s been your biggest barrier? To kind of learn from others that have gone before is the best way to kind of understand is this something we could potentially look at.”* (Transplant staff with 1–5 yrs of experience)	Facility currently uses an electronic referral program for kidney transplant	Concordance and Expansion: Interviews and surveys conveyed that most respondents used an electronic referral program. Interview data further explained what factors facilitated program use.

	Method of first learning about electronic referral program at dialysis facility or transplant center^b^	Concordance and Expansion: Survey and interview data described managerial staff and in-service training as the primary methods of how staff first learned about electronic referral programs at their dialysis facility or transplant center. Interviewees elaborated on this to describe how influential it was for managerial staff to promote these programs and provided more detail on the pros and cons of training.

	Reasons for nonuse among respondents that do not use an electronic referral program at their facility (*n* = 21)^c^	Concordance: Survey data described barriers to program use that aligned with program use barriers cited by interview participants.

	Reasons why a specific (ranked) electronic referral program is used the most^c^	Expansion: Interviewees expanded on the survey findings and highlighted specific examples of facilitators of their program's use.

*Theme 2:* A perceived imbalance between program training costs and gains in efficiency is a barrier to program use *“Just the unknown…not knowing how efficient and effective a program like this would be…so there may be some resistance too—would it be cost effective? Would we get a return on our investment for buying or purchasing, whatever they would have to do to use (electronic referral system)? But the main thing is just not knowing how effective it would be.”* (Dialysis staff with 1–5 yrs of experience)	How often respondents experienced issues with failed faxed referrals	Concordance and Expansion: Surveys and interview data indicated frustration with traditional methods of referral and communication (e.g., fax). Interviews expanded on survey findings by describing how frustration from alternative referral and communication methods may not be sufficient motivation to instigate the implementation of electronic referral programs because of other barriers or sources of hesitation.

	Reasons why a specific (ranked) electronic referral program is used the least.^c^	Concordance and Expansion: Interviewees echoed survey findings that described undesirable features of electronic referral programs that led to their disuse. Interviews expanded upon these barriers by describing how they compared with the perceived benefits of implementing the programs.

	Methods of electronic referral program training received by respondents^b^	Expansion: Interview data more fully described how electronic referral program training was enacted and how it was desired. Interviewees provided detailed information into the cost-benefit analysis dialysis facilities and transplant centers conducted when considering implementing electronic referral programs and their accompanying training.


★ = significant difference between dialysis and transplant staff responses using a chi-square or Fisher’s Exact test at α = 0.05.

a Values may not sum up to the total sample size (*n* = 110) because of missing data. There were < 10% missing data across variables.

b The survey question was a “check all that apply” format, allowing respondents to select multiple answers or none if applicable.

c The survey question was open-ended and underwent a content analysis to yield the displayed categories.

#### Reach Interview Results

*Theme 1: Facilitators of the program* *use are multilevel and include buy-in and endorsement from leadership, availability of program training, demonstrations of program value, and hands on interaction with the program.*

Interview participants described multilevel facilitators of electronic referral program use. Program use was facilitated by leadership buy-in and endorsement (i.e., mandates and workplace culture). Program training was also a facilitator, which included communication of the program’s value via colleagues, data, or self-discovery from hands-on interaction with the program (Table 3).

*Theme 2: A perceived imbalance between program training costs and gains in efficiency is a barrier to program use.*

A major barrier to program use identified was a perceived imbalance between training costs and efficiency gains. Participants voiced that the significant monetary and time investment to learn and implement a new electronic referral program was difficult to justify when efficiency gains are not immediately realized. Dialysis staff were more concerned than transplant staff about the immediate workload burden associated with these programs. Ultimately, uncertainty of the “return on investment” was a barrier to initial implementation of programs (Table 3).

#### Reach Mixed Methods Integration

Survey and interview findings supported the idea that managerial and collegial influence strongly motivated electronic referral program use. Both datasets highlighted perceived difficulty in using such programs because of limited usability or uptake by colleagues. Survey results were expanded by qualitative data, which added context to program uptake and use (Table 3).

### Effectiveness

#### Effectiveness Survey Results

Survey respondents found the programs easy to use (*n* = 77, 74.8%), effective for communicating with dialysis and transplant center staff (*n* = 70, 68.6%), and more efficient for sending or receiving referrals than other methods (*n* = 83, 81.4%). About half of the participants indicated that the programs assist the staff with knowledge of patients’ status in the kidney transplant process (*n* = 52, 50.5%), communicating with patients the outstanding issues stalling their progress (*n* = 47, 45.6%), and helping patients move more efficiently through the transplant process (*n* = 48, 47.1%). Dialysis staff were more likely to agree that their use of electronic programs improves their workflow than transplant staff (70.1% vs. 58.5%) (Table 4).

**Table 4:** Effectiveness: Survey and interview findings joint display

Qualitative theme and illustrative quotation	Quantitative survey item^a^^,^^b^	Meta-inferences
*Theme 3*: Electronic referral programs streamline the referral and transplant process, but their efficiency is limited by inconsistent bidirectional staff use of the program *“The only way that it’s gonna work well is if both parties want to use it. You know…if the other party is like, ‘Yeah, yeah. Whatever,’ but not utilizing to its fullest, then it’s just not. You might as well just not even bother.”* (Dialysis staff with > 10 yrs of experience)	The electronic referral program is easy to use	Concordance and Expansion: Survey and interview data suggested that electronic referral programs were relatively easy to use for dialysis and transplant staff. Interviewees further explained why these programs were easy to use and what about them could be difficult.

	The electronic referral program is an effective method for sending/receiving kidney transplant referrals	Concordance: Interview and survey data suggested that electronic referral programs were considered effective for sending/receiving transplant referrals.

	The electronic referral program is an effective method for communication with staff at transplant centers/dialysis facilities	Concordance: Interview and survey data suggested that electronic referral programs were considered effective for communicating with staff at transplant centers and dialysis facilities.

	The electronic referral program has improved communication between my dialysis facility and transplant centers	Discordance and Expansion: Survey respondents suggested that staff largely perceived electronic referral programs as helpful in improving communication between dialysis and transplant staff. However, interviewees described how the program alone was not always enough to improve communication but required consistent and dedicated use by both end-users. Interview data further explained how bidirectional use of these electronic referral programs was necessary but not always available, thereby rendering these programs no more effective for communication than traditional methods.

	The electronic referral program is more efficient for sending/receiving transplant referrals than other methods (e.g., faxing referrals)	Concordance**:** Survey and interview data agreed that electronic referral programs were less time-consuming for dialysis and transplant staff to use and the process of sending/receiving transplant referrals was less onerous than traditional methods.

	The electronic referral program improves my workflow	Discordance and Expansion: Survey data suggested that most respondents felt improvements in their workflow because of their electronic referral program, but interview data suggested that improvements were only felt with proper bidirectional use of the program between dialysis facilities and transplant centers. Interview data explained that staff workflow could be streamlined and improved with electronic referral programs but was dependent on consistent use between dialysis and transplant staff.

	The electronic referral program helps transplant centers/dialysis facilities understand where patients are in the transplant process	Discordance and Expansion: Survey respondents were approximately evenly split in their opinions about the utility of electronic referral programs to help them understand where patients are in the transplant process. Interviewees explained that these programs were indeed helpful in tracking patients, but were most efficient when they were regularly updated by both dialysis and transplant staff; so the information was accurate and current.

	The electronic referral program helps me communicate with patients who have outstanding issues preventing them from finishing their transplant evaluation (e.g., provide reminders about upcoming medical tests)	Discordance and Expansion: Almost half of survey respondents indicated that electronic referral programs could be used to communicate with patients with outstanding issues. However, interviewees suggested that staff perceived these programs to be ill-equipped to communicate directly with patients because they did not offer patient interfaces. Further, interview data described the desire from staff for these programs to incorporate patient interfaces to facilitate direct communication and to allow patients to track their own transplant process.

	The electronic transplant referral program helps patients more efficiently move through the transplant referral and evaluation process	Discordance and Expansion: A minority of survey respondents indicated that electronic referral programs did not help patients move more efficiently through the transplant process. In interviews, participants more often voiced that these electronic programs were not a means of moving patients through the process more quickly than traditional referral methods. Many participants expressed that although these programs may streamline referral workflows, they cannot hasten patients’ ability to complete necessary transplant requirements and move through the transplant process.


★ = significant difference between dialysis and transplant staff responses via a chi-square or Fisher’s Exact test at α = 0.05.

a Values may not sum to the total sample size (n=110) because of missing data. There were < 10% missing data across variables.

b Results reflect participants who responded “strongly agree” or “agree” to each survey item.

#### Effectiveness Interview Results

*Theme 3: Electronic referral programs streamline the referral and transplant process; however, their efficiency is limited by inconsistent bidirectional staff use of the program.*

Participants perceived electronic referral and communication programs to be highly effective at creating and updating transplant referrals and communicating with colleagues. The programs were generally deemed more efficient than other referral methods because they centralized patient information and streamlined workflow, which led to more and faster referrals. In addition, programs enabled direct and instantaneous messaging between dialysis and transplant staff, thereby facilitating easier and more frequent communication. However, participants found that these benefits were only realized when there was bidirectional use of the programs by dialysis and transplant staff. Many participants felt that electronic referral programs lost efficacy and efficiency when their counterparts did not use the same program, used it less than desired, or did not fully use its features. Bidirectional frustrations differed by role, with transplant staff citing incomplete referrals from dialysis facilities, and dialysis staff citing delayed or inconsistent communication from transplant centers. Overall, inadequate bidirectional use made these programs seem no better than previous methods (i.e., fax), where participants felt longer delays in a fragmented referral process (Table 4).

#### Effectiveness Mixed Methods Integration

Survey and interview data showed concordance in participants’ perceptions of electronic referral programs being effective and efficient for sending or receiving referrals and communicating across dialysis and transplant centers. However, survey respondents were less convinced of their effectiveness in monitoring and moving patients through the kidney transplant process, whereas interview participants emphasized their value—especially compared with tedious methods such as fax and phone calls. Interview findings expanded upon surveys to provide deeper insight into program limitations, such as their lack of direct communication with patients and their reliance on consistent bidirectional use to achieve superior efficiency (Table 4).

### Adoption

#### Adoption Survey Results

Among respondents who claimed their facility used an electronic referral program, a majority reported currently using those programs (*n* = 95, 94.1%). Most respondents overall reported using these programs to send or receive kidney transplant referrals (*n* = 75, 73.5%); however, more dialysis staff reported using these programs more than other communication methods (70.1% vs. 42.3%). Although most respondents agreed that their facility manager encourages staff to use their program (*n* = 63, 61.2%), only 13.7% (*n* = 14) reported that their manager mandates the program. Most respondents agreed that their coworkers use electronic referral programs to send or receive referrals and communicate with other centers (*n* = 66, 64.7%), and 68.6% (*n* = 70) agreed that they and their coworkers use these programs to manage patients’ kidney transplant evaluation processes (Table 5).

**Table 5:** Adoption: Survey and interview findings joint display

Qualitative theme and illustrative quotation	Quantitative survey item^a^	Meta-inferences
*Theme 4*: Staff who are trained to use electronic referral programs generally approve of and prefer them, but staff desire that more roles to be trained in using these programs to improve patient care and hold employees accountable *“The charge nurse, maybe theperitoneal dialysisnurse, the facility administrator, the assistant administrator—anyone that has patients and they need to find out where they are in the transplantation process—it would benefit them to know how to navigate [electronic referral platform].”* (Dialysis staff with 1–5 yrs of experience)	My facility currently uses an electronic referral program^b^	Concordance**:** Survey and interview data aligned and suggested that most participants used an electronic referral program in their dialysis facility or transplant center.

	Which method do you use the most to send/receive transplant referrals?	Concordance: Survey and interview data indicated that electronic referral programs were the most commonly used methods of sending/receiving transplant referrals in our sample.

	My facility uses the electronic referral program to communicate with transplant centers more than other methods (e.g., phone calls, email, fax)^b^	Concordance**:** Survey and interview data indicated that participants preferred using electronic referral programs for communicating with dialysis facilities and transplant centers rather than other methods.

	My facility supervisor encourages my co-workers and me to use the electronic referral program^b^	Expansion: Interview data more fully described the impact of managerial staff encouraging and endorsing the use of electronic referral programs. Interviews revealed that when supervisors encouraged use of electronic referral programs, it improved the work culture around those programs and facilitated their use. Interviews expanded on this topic to describe the desire for more staff to be encouraged to use these programs.

	My facility supervisor requires that I use the electronic referral program	Concordance and Expansion: Interview data agreed with survey data suggesting that though use of electronic referral programs was not often required, having their use encouraged by supervisors was equally as motivating. Interview data suggested that supervisors could foster a work culture centered around the use of electronic referral programs simply by endorsing and encouraging them rather than strictly mandating them.

	My coworkers who send/receive transplant referrals and/or communicate with dialysis facilities/transplant centers use the electronic referral program^b^	Concordance**:** Most survey and interview participants indicated that their coworkers used electronic referral programs for sending/receiving transplant referrals.

	My coworkers and I use the electronic transplant referral and communication program to help manage the transplant evaluation process^b^	Concordance**:** Most survey and interview participants indicated that their coworkers used electronic referral programs to manage the transplant evaluation process for their patients.


★ = significant difference between dialysis and transplant staff responses via a chi-square or Fisher’s Exact test at α = 0.05.

a Values may not sum up to the total sample size (*n* = 110) because of missing data. There were < 10% missing data across variables.

b Results reflect participants who responded “strongly agree” or “agree” to each survey item.

#### Adoption Interview Results

*Theme 4: Staff who are trained to use electronic referral programs generally approve of and prefer them; however, staff desire for more roles to be trained in using these programs to improve patient care and hold employees accountable.*

Participants generally found electronic referral programs efficient, helpful, and user-friendly; however, they recognized greater potential if more staff were trained on them. Many participants cited social workers in both dialysis and transplant settings as being the primary users of electronic referral programs, and dialysis staff especially voiced that other roles should be trained to use these programs to avoid overburdening social workers. Overall, staff suggested that expanding training could improve patient outcomes, enhance workflow efficiency, and increase accountability among staff. Electronic referral programs were described as efficient, easy to use, and capable of improving the acceptability of completing referrals and communicating with other dialysis or transplant staff. Exposure to the programs often led to a preference for incorporating them into daily workflows (Table 5).

#### Adoption Mixed Methods Integration

Survey and interview data supported the idea that participants who were exposed to electronic referral programs typically preferred them over other methods of referral or communication. In addition, data integration expanded the understanding of program adoption regarding the role of managerial influence and desired directions for staff use. Participants indicated that broader staff adoption of these programs could lead to a cultural shift, where their use becomes expected, which, in turn, could increase their potential to positively impact patients (Table 5).

### Implementation

#### Implementation Survey Results

Overall, most respondents agreed that they felt confident in their ability to use their electronic referral program (*n* = 84, 82.4%). However, dialysis staff more often agreed that their facility uses all of their program’s features as intended than transplant staff (70.1% vs. 28%). Most respondents agreed that the training for their program was easy to understand (*n* = 74, 71.8%), and they have been satisfied with ongoing program training (*n* = 61, 59.8%). Fewer transplant staff agreed that their program was not a burden on their facility than dialysis staff (48.0% vs. 80.3%). Most respondents reported “often” or “always” using electronic referral programs to communicate with dialysis or transplant centers (*n* = 67, 65.7%) and send or receive referrals (*n* = 85, 83.4%). Slightly fewer respondents reported using the programs to update dialysis or transplant centers during the evaluation process (*n* = 58, 56.3%), and this did not significantly vary by staff type. However, respondents differed in their frequency of logging into the programs; transplant staff most often reported logging in < 1 time/wk (*n* = 13, 50%), whereas dialysis staff most often reported logging in 2 to 4 times/wk (*n* = 31, 40.3%). The most frequently reported categorized barrier to program use was “I don’t have all the patient or facility information I need” (*n* = 32, 29.1%). Respondents least liked that the programs were not used enough by dialysis or transplant centers (*n* = 32, 36.8%), and most liked that they were easy to use and efficient (*n* = 39, 44.8%). For program improvement, participants wanted increases in corresponding dialysis or transplant centers’ use of the programs (*n* = 41, 49.4%) (Table 6).

**Table 6:** Implementation: Survey and interview findings joint display

Qualitative theme and illustrative quotation	Quantitative survey item^a^	Meta-Inferences
*Theme 5:* Staff believe that they know and use most of the electronic referral programs’ available features, but they appreciate continuous education or job aids to learn more or refresh their knowledge *“Yes, I think we use it as intended. I think as with most technology applications, I'm sure that there are features that we don't maximize the benefit of, but I do think overall we do use the program as it was built to be used.”* (Transplant staff with 1-5 years of experience)	I am confident about my ability to use the electronic referral program^b^	Concordance: Most survey and interview participants indicated that they felt confident in their ability to effectively use their electronic referral program.

	The training and onboarding for the electronic referral program was easy to understand^b^	Concordance: Survey and interview data suggested that the training for electronic referral programs was appropriate and acceptable to dialysis and transplant staff.

	The training and onboarding of dialysis facilities/transplant centers by the electronic transplant referral and communication system is not a burden on our facility^b^	Concordance and Expansion: Survey and interview data suggested that training for electronic referral programs was not onerous to dialysis facilities and transplant centers. Interview data expanded on this by describing how the training paid off in the end by enabling the use of these more efficient programs.

	On average, how many min do you spend training and providing support to dialysis/transplant providers on how to use the electronic referral program?	Concordance: Survey and interview data aligned as most participants described minimal time commitments in providing training support on how to use electronic referral programs.

	I have been satisfied with the ongoing training and technical support provided by the electronic referral program^b^	Concordance and Expansion: Survey and interview data suggested that staff were satisfied with ongoing training and support for electronic referral programs. Interviewees explained that they desired more refresher courses and continued education to ensure they continue to use their programs effectively.

	My facility uses the electronic referral program to its fullest potential^b^	Concordance: Survey and interview participants largely perceived their dialysis facility or transplant center to use their electronic referral program to its fullest potential. Both data sources suggested that staff believed that some potential remained to improve dialysis facility and transplant centers’ effective use of programs.

	I use all the electronic referral program features as intended by the program^b^	Concordance and Expansion: Survey data indicated that about half of participants used all electronic program features as intended which indicated room for improvement to utilize all features. Interviewees expanded on survey data to explain what training is desired to be able to use more features as intended by the programs.

*Theme 6:* Though some staff experienced a delay in their appreciation of electronic referral programs because of the steep learning curve required to master the programs, many prefer their electronic referral program to other methods of referral or communication that are more common yet less efficient. *“I think in general change is hard. And people have been doing things a certain way for a really long time, and that way has been working to an extent… That’s what we’re used to. And, you know, kind of getting people to understand why we’re making a change, why we’re doing something differently…we do have some staff that struggles with new software applications and so learning a new system and how to navigate a new system has been a challenge for some of our staff. Although when they finally bit the bullet and try that, we’ve gotten a lot of reports of success.”* (Dialysis staff with 10+ years of experience)	Frequency of use of electronic referral program for communication with transplant centers/dialysis facilities	Concordance: Survey and interview data agreed that staff used electronic referral programs to communicate with dialysis facilities and transplant centers the majority of the time.

	Frequency of use of electronic referral program to send/receive a referral for transplant	Concordance: Survey and interview data agreed that staff used electronic referral programs to send/receive transplant referrals the majority of the time.

	Frequency of use of electronic referral program to update transplant centers/dialysis facilities during the evaluation process	Concordance: Survey and interview data indicated that staff used electronic referral programs to update dialysis facilities and transplant centers relatively frequently, though they also agreed that there was a sizeable proportion that did not utilize the programs for this purpose.

	Frequency of logging into the electronic referral program	Concordance: Survey and interview data aligned in their indication that staff reported using electronic referral programs most often on a daily or weekly basis.

	Average percentage of transplant referrals made via the electronic referral program	Concordance and Expansion: Survey and interview data suggested that electronic referral programs were the most common method of transplant referral for our sample. Interview data revealed that certain limitations (i.e., the lack of electronic program use by a corresponding dialysis facility or transplant center) inhibited use of these programs for referral and required use of other traditional methods.

	Average time to refer/ process a referral using the electronic referral program	Concordance: Survey and interview data suggested that dialysis and transplant staff were able to refer or process a referral in a short timeframe (< 20 min).

	Average time spent using the electronic referral program to update a transplant center/dialysis facility about a patient's status during the evaluation process	Concordance: Survey and interview data suggested that updating a dialysis facility or transplant center using an electronic referral program was very quick (< 10 min).

	Documenting in the electronic referral program is too time consuming^b^	Concordance: A small proportion of survey responses indicated that documenting in the electronic referral program was too time-consuming, and this was reiterated in interviews where participants often cited the speed of the programs as one of its main benefits.

	Barriers to using the electronic referral program^c^	Concordance and expansion: Interview data provided an expanded understanding of barriers to using electronic referral programs. Interviewees reiterated barriers listed in surveys and provided more detail into why these barriers existed and how they influenced use of electronic referral programs.

	I have experienced problems when trying to use the electronic referral program^b^	Concordance and Expansion: Relatively few survey and interview participants indicated experiencing problems with electronic referral programs. Interviewees who did experience problems explained what those issues were and potential solutions that could remedy them.

	Characteristics liked least about electronic referral programs^c^	Expansion: Interviewees provided more detail about flaws with electronic referral programs and their least desirable traits. Interviewees voiced why these flaws were negative and how they reduced the efficacy and usability of the electronic referral programs.

	Characteristics liked most about electronic referral programs^c^	Expansion: interview data enriched understanding about the most desirable traits of electronic referral programs. Interviewees explained why these traits were helpful and how they impacted workflow efficiency.

	Suggested improvements to electronic referral programs	Concordance and Expansion: Interview data reiterated desired changes to improve electronic referral programs indicated in surveys. Interviewees explained why these changes were necessary to improve electronic referral programs and how they could improve their effectiveness and sustainability.


★ = significant difference between dialysis and transplant staff responses via a chi-square or Fisher’s Exact test at α = 0.05.

a Values may not sum up to the total sample size (*n* = 110) because of missing data. There were < 15% missing data across variables.

b Results reflect participants who responded “strongly agree” or “agree” to each survey item.

c The survey question was open-ended and underwent a content analysis to yield the categories displayed.

#### Implementation Interview Results

*Theme 5: Staff believe that they know and use most of the electronic referral programs’ available feature; however, they appreciate continuous education or job aids to learn more or refresh their knowledge.*

Participants voiced that they had adequate training to use their programs and expressed confidence with most program features; however, many participants indicated a desire to receive continuing education. Participants described using many different features of electronic referral programs in their daily workflow and using the programs because they believed they were intended to be used. Program training was found to be useful and easy to understand, and the most popular training approaches included virtual training, hands-on interaction in a practice environment, and live demonstrations by colleagues or program representatives.

*Theme 6:* *Though some staff experienced a delay in their appreciation of electronic referral programs because of the steep learning curve required to master the programs, many prefer their program to other methods of referral or communication that are more common yet less efficient.*

The transition to using electronic referral programs led to some hesitation from participants about learning and implementing a new program in their workflow. Participants expressed fear of the unknown that came with changing referral and communication protocols and were concerned that it may lead to more work and difficulty without benefit. Though the transition period was described as difficult by some, both dialysis and transplant staff found the learning curve to be worth it, as they recognized the programs’ gains in efficiency compared with slower, more error-prone methods such as fax and phone calls, especially when there was proper bidirectional use between dialysis and transplant staff (Table 6).

#### Implementation Mixed Methods Integration

Concordance was found in integration regarding participants’ positive perceptions of their confidence and fidelity to their program. In surveys and interviews, participants reported satisfaction with program training in their ease and variety. However, there was expansion of the data in interviews because participants voiced their desire for continuing education so they could stay current on program features. Integration yielded insights into the amount of time spent corresponding with dialysis and transplant centers and perceived repercussions from lack of bidirectional use of these programs. Survey data were further expanded as interview data revealed participants’ hesitancy to implement these programs and adopt training that may add to their busy schedules for unknown benefits (Table 6).

### Maintenance

#### Maintenance Survey Results

Most respondents reported preferring electronic programs to other methods of handling kidney transplant referrals (*n* = 76, 73.8%), agreed they would recommend the programs to colleagues (*n* = 73, 71.6%), and agreed that the programs were part of their regular work routine (*n* = 84, 82.4%). However, for each measure, agreement was significantly higher among dialysis staff than transplant staff (84.4% vs. 42.3%, 80.3% vs. 46.2%, and 92.2% vs. 52.0%, respectively). About one-third of respondents reported that they use the program more now than they did when it was first implemented at their facility (*n* = 40, 38.8%), and 80.4% of respondents (*n* = 82) agreed that they plan to use the program for future referrals, though again, dialysis staff were more in agreement than transplant staff (88.3% vs. 56.0%). A majority of respondents agreed that electronic referral programs help patients get kidney transplants (*n* = 62, 60.8%) and that their facility will likely continue using their program in the future (*n* = 81, 79.4%). Alternatively, 33.3% of all respondents agreed that the program needs substantial improvements to be effective at their facility (*n* = 34) (Table 7).

**Table 7:** Maintenance: survey and interview findings joint display

Qualitative theme and illustrative quotation	Quantitative survey item^a^	Meta-inferences
*Theme 7:* Integration with electronic medical records and more strict data requirements for electronic referral programs are desired changes that would improve the sustainability of such programs *“To us, the easiest way to communicate would be through [electronic medical record system], you know, because we use [electronic medical record system], so I guess that would be something that if [electronic medical record system] and [electronic referral platform] could talk to each other…that's probably the best way, because…the answer to all those questions are in [electronic medical record system], usually, you know?…To have those two systems communicate that would be helpful.”* (Transplant staff with 6-10 years of experience)	I prefer the electronic referral program to other methods of making/receiving transplant referrals (e.g., faxing referrals, phone calls)^b^	Concordance**:** Survey and interview data suggested that most staff preferred their electronic referral program to other methods of making or receiving transplant referrals.

	I would recommend the electronic referral program to colleagues at other facilities^b^	Concordance**:** Survey and interview data agreed that most staff were satisfied enough with their electronic referral program to recommend it to their colleagues at other dialysis facilities or transplant centers.

	Using the electronic referral program is now part of my regular work routine^b^	Concordance**:** Survey and interview participants indicated that electronic referral programs were regular parts of dialysis and transplant staff’s work routines.

	Current use of electronic referral program compared with use at first implementation	Expansion: Interview data revealed why participants reported using their electronic referral program more or less now than when it was first implemented. Interviewees also explained why they have used the program consistently over time and what could cause that to shift in the future.

	I plan to use the electronic referral program in the future when sending/receiving transplant referrals.^b^	Concordance: Survey and interview data suggested that most participants planned to continue using their electronic referral program in the future.

	The electronic referral program needs substantial improvements to be more effective at my facility.^b^	Expansion: Interview participants expanded on the perspectives from survey respondents that suggested changes were necessary to improve electronic referral program effectiveness. Interviewees provided details about what those changes could be and how they could improve program effectiveness.

	Changes are needed to the electronic referral program to make it something I will use regularly.^b^	Expansion: Interview participants provided detailed information about why changes were needed to make electronic referral programs more sustainable and regularly used. Interviewees suggested what those changes could be and how they could improve program use and long-term sustainability.

	Using programs like the electronic referral program will help patients get kidney transplants.^b^	Concordance and Expansion: Survey and interview data cohesively suggested that electronic referral programs can help patients receive kidney transplants. Interview data more thoroughly described how these programs can improve communication between dialysis and transplant staff and hasten the kidney transplant process for patients.

	My facility will likely continue using the electronic referral program in the future.^b^	Concordance: Survey and interview data suggested that dialysis facilities and transplant centers are likely to continue using electronic referral programs in the future.


★ = significant difference between dialysis and transplant staff responses via a chi-square or Fisher’s Exact test at α = 0.05.

a Values may not sum to the total sample size (*n* = 110) because of missing data. There were < 10% missing data across variables.

b Results reflect participants who responded “strongly agree” or “agree” to each survey item.

#### Maintenance Interview Results

*Theme 7: Integration with electronic medical records (EMRs) and more strict data requirements for electronic referral programs are desired changes that would improve the sustainability of such programs.*

Participants who were interested in continuing to use electronic referral programs provided suggestions for adapting these programs. Primarily, participants wanted more integration of electronic referral programs with EMR systems to reduce manual data entry and task redundancy. Participants identified a need for more rigorous data entry protocols (e.g., mandating certain fields). Transplant staff voiced discontent with data inconsistencies between dialysis and transplant center users. Similarly, participants desired consistency in program use so that more centers can get on board with a specific program and adhere to uniform expectations (Table 7).

#### Maintenance Mixed Methods Integration

After integration, concordance was found in the idea that many participants who used electronic referral programs preferred them in their workflow and desired to continue using them. Survey data suggested that there was room for improvement in these electronic referral programs, and interview data expanded that by describing what adaptations could be made to these programs to encourage their future use. Interview data suggested that integration with EMRs and having more strict and uniform data requirements would promote continued use and potential future adoption of electronic referral programs (Table 7).

## Discussion

This mixed-methods process evaluation provides insight into the implementation and use of electronic kidney transplant referral and communication programs among dialysis and transplant center staff in the Southeastern US. Electronic referral programs had a wide reach in this population, were implemented with moderate fidelity, and were generally viewed as more usable and efficient than paper- or fax-based referral methods, supporting their potential for continued use. Although perceptions were generally positive across staff types, dialysis staff tended to report more favorable views and higher use than transplant staff, though both groups offered complementary perspectives on barriers, facilitators, and opportunities for future improvement. Like previous process evaluations of interventions in kidney failure treatment settings,^27^^,^^28^ this study highlights contextual factors that shape the uptake of initiatives intended to improve access to kidney transplant.

Despite widespread adoption, incomplete bidirectional use between dialysis and transplant staff and limited EMR integration led to frustration and reduced perceived effectiveness, sustainability, and utility of these programs for supporting coordinated care. This is consistent with previous research that identified a communication gap between dialysis and transplant centers as a barrier to quality patient care.^13^ When well-integrated into EMR systems, electronic referral programs may help bridge this gap by improving communication and tracking of patients’ progress, ultimately improving transparency and efficiency throughout the transplant process.

Leadership buy-in and endorsement featured prominently in our study and, consistent with previous research, was associated with improved intervention uptake and fidelity.^27^ In this study, many respondents reported being encouraged to use their program, but fewer were mandated to use it over other forms of referral. Stronger motivation from dialysis and transplant leadership could facilitate a positive culture around electronic referral programs and improve uptake. However, as electronic referral programs continue to evolve, broader system-level dynamics—such as preferences for specific platforms—and subsequent health system compatibility issues warrant attention because they may shape accessibility and consistency of program use across centers.

Adequate staff education and training emerged as important factors influencing program implementation. Respondents valued live demonstrations and continuing education for electronic referral programs, noting that limited understanding of program benefits was an implementation barrier because it reduced motivation to invest time in adopting new programs. Consistent with other studies, social workers were the most common role to be trained on and use electronic referral programs because of their central role in transplant-related education.^29^^,^^30^ Despite social workers being most involved in managing patients’ transplant processes, participants, especially dialysis staff, indicated that additional roles should be trained on such programs to avoid overburdening social workers. Evidence suggests that having more staff roles trained on an intervention is associated with increased fidelity.^27^ Expanding training beyond social workers may therefore enhance uptake and sustained use of electronic referral programs in dialysis and transplant centers.

Although participants described being able to observe latent benefits of electronic referral programs within their own centers, evidence of their broader effectiveness in improving access to kidney transplantation is still lacking. Center-level monitoring offers an opportunity to generate and share data on program performance with prospective adopters; however, our findings underscore that the wider implications of these programs remain unknown. Our data do not establish whether electronic referral programs increase referral volume or improve downstream outcomes like evaluation initiation or completion, waitlisting, or transplantation. Even if these programs led to increased referral throughput, it may not translate into improved access and could exacerbate existing bottlenecks if transplant centers cannot absorb higher referral volumes. These programs may nonetheless offer benefits beyond referral initiation, including improved communication, transparency, and patient tracking. Such features could alleviate workflow delays, strengthen coordination between dialysis and transplant centers, and benefit patients by moving them more seamlessly through the transplant process. Future research should evaluate how electronic referral programs influence patient-centered outcomes and system-level performance.

Several limitations of this mixed methods implementation study merit discussion. First, data were self-reported and may be subject to response and recall bias. In addition, perspectives from staff who did not use electronic referral programs were not captured, which limited our insight into their centers’ barriers to program adoption. Further, participants used different electronic referral and communication platforms with varying functionality (e.g., EMR integration), and many survey respondents were unsure about how long their programs had been in place (52.7% missing or “I don’t know” responses), limiting information on program context and duration of use. Selection bias is also possible if respondents differed from staff who were not involved. Because this study was conducted solely within the Southeastern US, the findings may not represent implementation experiences nationwide, especially because geographic variability in dialysis practices, transplant referral patterns, and resource availability was not accounted for in this study. Finally, our sample included more dialysis than transplant staff, though study credibility was improved by the large representation of social workers, who are primary users of these programs. Combining quantitative and qualitative data enhances the robustness of our findings and contributes to the growing body of implementation science literature in nephrology and kidney transplant.^31^

## Conclusion

This research provides guidance for dialysis and transplant centers considering implementing electronic referral programs by emphasizing the importance of demonstrating the value of such programs, providing effective training methods, and using staff-defined motivators for uptake. This guidance is especially relevant given the potential for these tools to meet recent prewaitlisting data collection recommendations from the Health Resources and Services Administration.^32^ In addition, developers of these programs can use the actionable recommendations from these results to inform future modifications to their programs to meet user needs. More broadly, this work suggests that widespread adoption of these programs may help to streamline workflows for dialysis and transplant staff; however, the extent to which increased electronic referrals translate into improved patient outcomes remains unknown. Future studies are needed to evaluate the effectiveness and downstream outcomes of electronic referral programs to ensure that their use leads to meaningful improvements in patient care and transplant access.

## Disclosure

MU reports serving as Co–Editor-in-Chief of the Journal of Nephrology Social Work, the Region 3 representative on the Organ Procurement and Transplantation Network’s Ethics Committee, a member-at-large on the American Society of Transplantation’s Psychosocial and Ethics Community of Practice, and Community Engagement Chair for the Southeastern Kidney Transplant Coalition. All the other authors declared no competing interests.

## Supplementary Material

Supplementary File (PDF) Table S1. COnsolidated criteria for REporting Qualitative research (COREQ) checklist. Table S2. Strengthening the Reporting of OBservational studies in Epidemiology (STROBE) checklist. Table S3. Checklist of mixed methods elements in a submission to advance the methodology of mixed methods research. Table S4. Reach, Effectiveness, Adoption, Implementation, and Maintenance (RE-AIM) Framework dimensions, measure in study, and example survey and semi-structured interview questions.

## Footnotes

Supplementary File (PDF)
**Table S1.** COnsolidated criteria for REporting Qualitative research (COREQ) checklist. **Table S2.** Strengthening the Reporting of OBservational studies in Epidemiology (STROBE) checklist. **Table S3.** Checklist of mixed methods elements in a submission to advance the methodology of mixed methods research. **Table S4.** Reach, Effectiveness, Adoption, Implementation, and Maintenance (RE-AIM) Framework dimensions, measure in study, and example survey and semi-structured interview questions.
