# Supplementary material for: Assessing the Implementation of Electronic Kidney Transplant Referral and Communication Programs
Source: Kidney Int Rep. 2026 Feb 10;11(5):106352. doi: 10.1016/j.ekir.2026.106352 (PMC13022656; doi:10.1016/j.ekir.2026.106352)
Supplement: Supplementary File (PDF) — Table S1. COnsolidated criteria for REporting Qualitative research (COREQ) checklist. Table S2. Strengthening the Reporting of OBservational studies in Epidemiology (STROBE) checklist. Table S3. Checklist of mixed methods elements in a submission to advance the methodology of mixed methods research. Table S4. Reach, Effectiveness, Adoption, Implementation, and Maintenance (RE-AIM) Framework dimensions, measure in study, and example survey and semi-structured interview questions. [file mmc1.pdf]

Supplementary Table S1. COnsolidated criteria for REporting Qualitative research (COREQ) Checklist[17]

Item No	Guide Questions/Description	Reported on Page #
**Domain 1: Research team and reflexivity**		
**Personal Characteristics**		
1. Interviewer/ facilitator	Which author/s conducted the interview or focus group?	N/A
2. Credentials	What were the researcher's credentials? E.g., PhD, MD	1
3. Occupation	What was their occupation at the time of the study?	N/A
4. Gender	Was the researcher male or female?	N/A
5. Experience and training	What experience or training did the researcher have?	1 and 7
**Relationship with participants**		
6. Relationship established	Was a relationship established prior to study commencement?	N/A
7. Participant knowledge of the interviewer	What did the participants know about the researcher? e.g. personal goals, reasons for doing the research?	N/A
8. Interviewer characteristics	What characteristics were reported about the interviewer/facilitator? e.g. Bias, assumptions, reasons and interests in the research topic	N/A
**Domain 2: study design**		
**Theoretical framework**		
9.Methodological orientation and Theory	What methodological orientation was stated to underpin the study? e.g. grounded theory, discourse analysis, ethnography, phenomenology, content analysis	6-8
**Participant selection**		
10. Sampling	How were participants selected? e.g., purposive, convenience, consecutive, snowball	5-6
11. Method of approach	How were participants approached? e.g., face-to-face, telephone, mail, email	5-6
12. Sample size	How many participants were in the study?	8
13. Non-participation Setting	How many people refused to participate or dropped out? Reasons?	8
14. Setting of data collection	Where was the data collected? e.g., home, clinic, workplace	5 and 7

15. Presence of nonparticipants	Was anyone else present besides the participants and researchers?	N/A
16. Description of sample	What are the important characteristics of the sample? e.g. demographic data, date	8, Table 1, Table 2
**Data collection**		
17. Interview guide	Were questions, prompts, and guides provided by the authors? Was it pilot tested?	6, Supplementary Table 4
18. Repeat interviews	Were repeat interviews carried out? If yes, how many?	N/A
19. Audio/visual recording	Did the research use audio or visual recording to collect the data?	7
20. Field notes	Were field notes made during and/or after the interview or focus group?	7
21. Duration	What was the duration of the interviews or focus group?	8
22. Data saturation	Was data saturation discussed?	6
23. Transcripts returned	Were transcripts returned to participants for comment and/or correction?	N/A
**Domain 3: analysis and findings**		
**Data analysis**		
24. Number of data coders	How many data coders coded the data?	7
25. Description of the coding tree	Did the authors provide a description of the coding tree?	N/A
26. Derivation of themes	Were themes identified in advance or derived from the data?	7
27. Software	What software, if applicable, was used to manage the data?	7
28. Participant checking	Did participants provide feedback on the findings?	N/A
**Reporting**		
29. Quotations presented	Were participant quotations presented to illustrate the themes/findings? Was each quotation identified? e.g., participant number	Tables 3-7
30. Data and findings consistent	Was there consistency between the data presented and the findings?	8-21
31. Clarity of major themes	Were major themes clearly presented in the findings?	8-17 and Tables 3-7
32. Clarity of minor themes	Is there a description of diverse cases or a discussion of minor themes?	N/A

Supplementary Table S2. Strengthening the Reporting of OBservational studies in Epidemiology (STROBE) Checklist[18]

STROBE Statement—Checklist of items that should be included in reports of ***cross-sectional studies***	**Item No.**	**Recommendation**	**Page No.**
**Title and abstract**	1	(*a*) Indicate the study's design with a commonly used term in the title or the abstract	1-2
		(*b*) Provide in the abstract an informative and balanced summary of what was done and what was found	2
**Introduction**			
Background/rationale	2	Explain the scientific background and rationale for the investigation being reported	4
Objectives	3	State specific objectives, including any prespecified hypotheses	4
**Methods**			
Study design	4	Present key elements of study design early in the paper	5
Setting	5	Describe the setting, locations, and relevant dates, including periods of recruitment, exposure, follow-up, and data collection	5
Participants	6	(*a*) Give the eligibility criteria, and the sources and methods of selection of participants	5-6
Variables	7	Clearly define all outcomes, exposures, predictors, potential confounders, and effect modifiers. Give diagnostic criteria, if applicable	6-7
Data sources/ measurement	8*	For each variable of interest, give sources of data and details of methods of assessment (measurement). Describe comparability of	6-7

		assessment methods if there is more than one group	
Bias	9	Describe any efforts to address potential sources of bias	NA
Study size	10	Explain how the study size was arrived at	8
Quantitative variables	11	Explain how quantitative variables were handled in the analyses. If applicable, describe which groupings were chosen and why	6-8
Statistical methods	12	(*a*) Describe all statistical methods, including those used to control for confounding	6-8
		(*b*) Describe any methods used to examine subgroups and interactions	NA
		(*c*) Explain how missing data were addressed	NA
		(*d*) If applicable, describe analytical methods taking account of sampling strategy	NA
		(*e*) Describe any sensitivity analyses	NA
**Results**			
Participants	13*	(a) Report numbers of individuals at each stage of study—eg numbers potentially eligible, examined for eligibility, confirmed eligible, included in the study, completing follow-up, and analysed	8
		(b) Give reasons for non-participation at each stage	8
		(c) Consider use of a flow diagram	NA
Descriptive data	14*	(a) Give characteristics of study participants (eg demographic, clinical, social) and information on exposures and potential confounders	8, Tables 1-2
		(b) Indicate number of participants with missing data for each variable of interest	8, Tables 1-7

Outcome data	15*	Report numbers of outcome events or summary measures	8-17, Table 1-7
Main results	16	(*a*) Give unadjusted estimates and, if applicable, confounder-adjusted estimates and their precision (eg, 95% confidence interval). Make clear which confounders were adjusted for and why they were included	NA
		(*b*) Report category boundaries when continuous variables were categorized	NA
		(*c*) If relevant, consider translating estimates of relative risk into absolute risk for a meaningful time period	NA
Other analyses	17	Report other analyses done—eg analyses of subgroups and interactions, and sensitivity analyses	NA
**Discussion**			
Key results	18	Summarise key results with reference to study objectives	17-20
Limitations	19	Discuss limitations of the study, taking into account sources of potential bias or imprecision. Discuss both direction and magnitude of any potential bias	20-21
Interpretation	20	Give a cautious overall interpretation of results considering objectives, limitations, multiplicity of analyses, results from similar studies, and other relevant evidence	17-21
Generalisability	21	Discuss the generalisability (external validity) of the study results	20-21
**Other information**			
Funding	22	Give the source of funding and the role of the funders for the present study and, if applicable,	21-22

		for the original study on which the present article is based	

*Give information separately for exposed and unexposed groups.

Note: An Explanation and Elaboration article discusses each checklist item and gives methodological background and published examples of transparent reporting. The STROBE checklist is best used in conjunction with this article (freely available on the Web sites of PLoS Medicine at http://www.plosmedicine.org/, Annals of Internal Medicine at http://www.annals.org/, and Epidemiology at http://www.epidem.com/). Information on the STROBE Initiative is available at www.strobe-statement.org.

Supplementary Table S3. Checklist of Mixed Methods Elements in a Submission to Advance the Methodology of Mixed Methods Research[19]

**Manuscript title**: Assessing Implementation Outcomes of Electronic Kidney Transplant Referral and Communication Programs in the Southeastern U.S.: A Mixed Methods Study	**Yes**	**No**	**Page number** (if not applicable, indicate NA)
**Title**			
1. Does the title directly indicate or sufficiently allude to the methodological contribution of the article?		X	NA
Abstract			
2. Does the abstract include an explicit statement about how the methodological contribution will advance an understanding of the chosen topic of the paper?	X		2
3. Does the abstract indicate the methodological/theoretical contribution of the study to the field of mixed methods research?		X	NA
**Main text of the article**			
4. Does the main text reiterate and expand on the methodological contribution as identified in the abstract?	X		4-21
5. Does the background contain a rigorous review and citations of relevant mixed methods literature to support examining the methodological aim?		X	NA
6. Does the background include an explicit methodological aim?	X		4
7. Does the background contain an explication of the article's structure and methodological points that will be addressed by the paper?	X		4
8. In the body of paper, are each of the points identified in Element 7 addressed with examples in the order specified?	X		4-21
9. In the discussion, are the explicit points made in Element 8 synthesized together to logically support the overarching methodological aim?	X		17-21
10. In the discussion section, is there a specific subsection "Contribution to the Field of Mixed Methods Research" that reviews the points made and extant literature to articulate the articles novel contribution(s) to mixed methods?		X	NA
11. Does the paper have a discussion of methodological limitations of the paper?	X		20-21
12. Does the paper have a clear writing style with sufficient headers and subheaders such that the reader can readily follow the flow and argumentation?	X		4-21

13. Have the references been cited according to the current American Psychological Association (APA) style?		X	NA
14. Does the paper include a figure or illustration to visualize the overarching concept of the manuscript?	X		Figure 2
15. Does the discussion section include recommendations for future mixed methods inquiry based on the paper's unique contribution or limitations?		X	NA
**Additional elements for empirical methodological articles only**			
16. Does the background of the paper include explicit statements of both the methodological purpose and purpose of the empirical study separately?		X	NA
17. Does the description of the methods include sufficient detail about the procedures used and present these in a logical order?	X		4-8
18. Does the submission include a procedural diagram of the data collection and analysis procedures as a figure?	X		Figure 2
19. Does the submission include a visual structure, or example, joint display, to illustrate integration and interpretation of the qualitative and quantitative findings?	X		Tables 3-7
20. Does the discussion articulate how the use of a mixed methods approach advanced a greater understanding of the substantive topic compared with using a monomethod approach?	X		20-21

Supplementary Table S4. Reach, Effectiveness, Adoption, Implementation, and Maintenance (RE-AIM) Framework Dimensions[20], Measure in Study, and Example Survey and Semi-Structured Interview Questions

**Dimensions**	**Measure**	**Example Questions [data collection source]**
Reach	Proportion of staff using an electronic transplant referral and communication program<br><br>How electronic program was first implemented in the dialysis/transplant facility including barriers and facilitators to uptake	Does your facility currently use an electronic program for communicating with transplant centers and/or making referrals for transplant (e.g., T-REX, TxAccess)? [survey]<br><br>How did you first learn about the [specific program]? [interview]
Effectiveness	Perceived effectiveness of the electronic program for transplant referrals and communication<br><br>Usability, acceptability, and efficiency of the electronic program	Please rate your agreement with the following statements: [survey]<br>• The electronic transplant referral and communication program is easy to use.<br>• The electronic transplant referral and communication program is an effective method for communication with staff at transplant centers/dialysis facilities.<br>• The electronic transplant referral and communication program improves my workflow.<br><br>How has using [specific program] impacted your workflow? [interview]<br><br>How has [specific program] impacted your ability to help patients move through the transplant process after being referred for transplant? [interview]
Adoption	How often and why dialysis and transplant staff use electronic programs in their typical workflow	Which method do you use the most to send/receive transplant referrals? [survey]<br><br>What strategies might encourage other staff at your facility to use [specific program]? [interview]
Implementation	Perceptions of electronic program training, frequency of electronic program use, fidelity to the electronic program, and	Please rate your agreement with the following statements: [survey]

	challenges and benefits to using electronic programs	• My facility uses the electronic transplant referral and communication program to its fullest potential.<br><br>What are some of the barriers to using the electronic transplant referral and communication program? (Please select all that apply) [survey]<br><br>Can you tell me about the training/onboarding you received on [specific program]? [interview]<br><br>How does [specific program] compare to other methods of communication with transplant centers? [interview]
Maintenance	Assessment of dialysis and transplant staff's intention to use their electronic program in the future and suggestions for adapting the program to be more acceptable	Please rate your agreement with the following statements: [survey]<br>• Using the electronic transplant referral and communication program is now part of my regular work routine.<br>• I plan to use the electronic transplant referral and communication program in the future when sending/receiving transplant referrals.<br><br>What would help your facility to continue using [specific program]? [interview]
